# Effect of a Combination of Fenugreek Seeds, Linseeds, Garlic and Copper Sulfate on Laying Hens Performances, Egg Physical and Chemical Qualities

**DOI:** 10.3390/foods8080311

**Published:** 2019-08-02

**Authors:** Besma Omri, Ben Larbi Manel, Zemzmi Jihed, Alessandra Durazzo, Massimo Lucarini, Raffaele Romano, Antonello Santini, Hédi Abdouli

**Affiliations:** 1 Laboratory of improvement and integrated development of animal productivity and food resources, Department of animal production, Higher School of Agriculture of Mateur, University of Carthage, 1054 Carthage, Tunisia; 2 National Agronomy Institute-Tunis, University of Carthage, 1054 Carthage, Tunisia; 3 Research Unit of biodiversity and resource development in mountain areas of Tunisia (UR17AGR14), Higher School of Agriculture of Mateur, University of Carthage, 1054 Carthage, Tunisia; 4 CREA—Research Centre for Food and Nutrition, Via Ardeatina 546, 00178 Rome, Italy; 5 Department of Agriculture, University of Napoli Federico II, Via Università 100, 80055 Portici (NA), Italy; 6 Department of Pharmacy, University of Napoli Federico II, Via D. Montesano 49, 80131 Napoli, Italy

**Keywords:** fenugreek seed, garlic, linseed, copper sulfate, yolk, cholesterol

## Abstract

Several investigations have suggested that fenugreek seeds may have a hypocholesterolemic activity, and thus be efficient in the treatment of egg yolk cholesterol. The objective of the current study was to evaluate the effect of dietary incorporation of 3% of fenugreek seed combined with 3% of linseed, 1% of garlic paste, and 0.078% of copper sulfate on laying performance, egg quality and lipids profile. Forty four, 41 weeks old, Novogen White laying hens received for 42 days 100 g/d of basal diet (control) or experimental diet (CFSGLSCS). With the exception of egg weight, which showed a significant increase for hens fed on CFSGLSCS with 57.99 g compared to 56.34 g for the control group, egg production (90.84% for control compared to 87.89% for experimental diet), egg mass (50.95 g/d for control compared to 50.87 g/d for CFSGLSCS), feed efficiency (1.94 for control compared to 1.98 for CFSGLSCS) were not affected by dietary treatments. The addition of CFSGLSCS reduced (*p* < 0.05) egg yolk cholesterol by 5.4% and blood cholesterol from 158.42 mg/dL to 122.82 mg/dL for control and CFSGLSCS, respectively. The dietary addition of CFSGLSCS increased (*p* < 0.05) total lipids from 4.5 g/egg to 5.23 g/egg and didn’t affect (*p* > 0.05) yolk triglycerides.

## 1. Introduction

In the perspective of functional foods and nutraceuticals [1,2,3,4,5], many researchers and egg producers are actually working together to produce a designer or multi-enriched egg. This egg is a source of high quality proteins, vitamins, and lipids, such as phospholipids and polyunsaturated fatty acids (PUFA) [6,7]. In fact, compared to the multi-enriched or designer egg, the standard egg contains from 183 mg/egg [8] to 386 mg/egg [9,10] of cholesterol and around 150 mg/egg of saturated fatty acids [11,12,13]. In this field, many studies have been made to decrease egg yolk cholesterol concentration [9,10,14] using natural plant products like garlic [15], fenugreek seeds [9,10,16] and copper [17,18]. Egg yolk polyunsaturated fatty acids enhancement using linseeds [19,20,21]. The effect of dietary incorporation of fenugreek seeds on egg cholesterol content has been inconsistent and the reduction was low not exceeding 1 mg/g egg yolk [16,20]. Dietary garlic paste (38 g/kg) reduced serum cholesterol by 23% in 12 weeks-old Leghorn pullets, when diets were fed for four weeks [22]. Egg yolk cholesterol was reduced by feeding of 10 or 30 g/kg garlic powder to laying hens for three weeks [23]. Concerning linseed, it is a very rich source of n-3 polyunsaturated fatty acids (PUFA) among many vegetable sources [24]. It is well documented that n-3 PUFA bring potential benefits for the human health. That is why, many researchers have focused on linseeds inclusion in the diets of layers to enhance the egg n-3 PUFA content [25,26,27,28]. In view of the above, the objective of the present study was to evaluate the effect of the dietary incorporation of fenugreek seeds combined with linseeds, garlic paste, and of copper sulfate on laying performance, egg quality, and lipids profile.

## 2. Materials and Methods 

### 2.1. Dish Preparation

Three kg of fresh fenugreek seeds were offered from the Higher School of Agriculture of Mateur, north of Tunisia and carefully cleaned from foreign matter. Three kg of linseed were purchased from a regional producer located at Mateur and cleaned from foreign matter. One kg of garlic was pelt, cut in a little fragment and mixed with distilled water. The composition of the basal diet was: 95 kg of basal diet were mixed with 5 kg of vegetable oil. The experimental diet contained: 2.9 kg of fenugreek seed, 2.9 kg of linseed, 76 g of CuSO_4_ and 970 g of garlic mixed with 92 kg of basal diet (CFSGLSCS).

### 2.2. Ethical Considerations

The experimental protocol was approved by the Official Animal Care and Use Committee of the Higher School of Agriculture of Mateur (protocol N^o^ 05/15) before the initiation of research and followed the Tunisian guidelines approved by the committee on care, handling, and sampling of the animals.

### 2.3. Experimental Design

Forty-four Novogen White laying hens aged 41 weeks were divided randomly into two treatment groups with 22 birds each. They were allocated each group to one of two dietary treatments: Basal diet and experimental diet. Each hen was daily fed 100 g of diet. The composition of the diets is shown in Table 1. Authors model some ingredients on other studies as follows:

Whole Fenugreek seeds at 2.9 kg equivalent to 3%. Fenugreek seed has been used in many previous researches to reduce egg cholesterol content [10,12,29,30]. However, it contains many bioactive compounds including powerful antioxidants that could prevent lipids oxidation, particularly in eggs enriched with polyunsaturated fatty acids. In this study, in order to reevaluate its effect on egg cholesterol content and fenugreek seed was included at 3% (2.9 kg) into the linseed supplemented diet. 

Garlic at 970 g equivalent to 1%. Many animal studies have suggested that garlic supplemented diets may inhibit the synthesis of cholesterol and fatty acids in the liver [31,32,33]. For example, Motamedi et al. [32] reported that dietary supplementation of 1% of garlic powder and of 1% of fenugreek powder reduced laying hens’ serum cholesterol from 242.00 mg/dL to 175 mg/dL. 

CuSO4 at 76 g equivalent to 0.078%. Copper supplementation to laying hen diets at pharmacological concentrations (>250 mg/kg) has been demonstrated to cause a reduction in egg yolk cholesterol content [34,35]. 

Linseeds at 2.9 kg equivalent to 3% [36,37]. Linseed is often used in the production of ω3-enriched eggs and some reports indicated that there was an increase in egg yolk unsaturated fatty acids concentrations when hens were fed yellow corn-soybean meal diets containing linseed at an incorporation rate of 5% [38]. It was found that the long-term use of linseed at an incorporation level of 10% increased the incidence of liver hemorrhages [21] presumably due to the oxidative rancidity of the accumulated long chain unsaturated fatty acids. 

Hens were housed in individual cages with individual feed-trough and common water-trough in a room with an ambient temperature of about 20 °C and a photoperiod of 16 h light: 8 h darkness cycle. Water was provided ad libitum intake throughout the trial period which lasted 42 days.

### 2.4. Data Collection

All birds were weighed individually at the beginning and the end of the experiment trial to determine the hens live weight changes. The feed was offered daily at 7:30 a.m. and refusal was measured on days 7, 14, 21, 28, 35 and 42 of the experiment assay. Egg production and weight were recorded daily. Daily feed consumption, hen-day laying rate (number of laid eggs ×100/number of feeding days) and feed efficiency (feed consumption/(number of eggs × egg weight)) were calculated per period (P1–P6) corresponding to the days 1–7, 8–14, 15–21, 22–28, 29–35 and 36–42. Two yolks of each egg laid during the 33rd and 34th days of the experiment assay were mixed (11 yolks/lots) and were used for the analysis of egg qualities (egg weight, shell weight, egg shell thickness, yolk weight and yolk cholesterol, triglycerides and total lipids).

Blood samples of nine hens/lot were collected at the end of the experiment (the 42nd day) from the brachial wing vein using sterilized syringes and needles and used for serum cholesterol determination after centrifugation at 3000 rpm for 15 min.

### 2.5. Chemical Analysis

The dry matter of the diets (DM) was determined at 105 °C for 24 h while all other analyses were done on samples dried at 65 °C and ground in a mill to pass through a 0.5 mm screen. Ash content was determined by igniting the ground sample at 550 °C in a muffle furnace for 12 h. The Association of Official Analytical Chemists method [40] was used for crude proteins (CP) determination. Neutral detergent fiber (NDF) was determined as described by Van Soest et al. [41], but sodium sulphite and alpha amylase were omitted from the NDF procedure. 

An enzymatic method (cholesterol enzymatic colorimetric test, CHOD-PAP, triglycerides enzymatic colorimetric GPO-PAP Biomaghreb, Ariana, Tunisia) were used for cholesterol in the serum and in egg yolk solubilized in 2% (*w*/*v*) NaCl solution [42]. 

Total lipids of the homogenized egg yolk was determined by extraction with isopropanol: hexane (30:70) (*v*/*v*) and then gravimetrically estimated. 

### 2.6. Statistical Analysis

Collected data were subjected to the analysis of variance using the General Linear Model (GLM) procedure of the Statistical Analysis System SAS [43]. Daily feed consumption, hen-daily laying rate, egg weight and mass and feed efficiency data were first tested for the diet, period and diet x period effects. Period and treatment x period effects were found to be not significant (α = 0.05) and, therefore, only treatment (diets) effect was retained. Multiple comparison tests were subjected to the Duncan test.

## 3. Results and Discussion

### 3.1. Laying Performance

Body weight changes, daily refusal, hen-day laying rate, egg mass and feed efficiency during the experimental assay are shown in Table 2. 

Although the feed was restricted to 100 g/hen/day, daily refusal and feed consumption, were both affected by dietary treatment (*p* < 0.05). Daily refusal was reduced (*p* < 0.05) for the experimental group from 0.62 g DM/day to 2.46 g DM/day for the control group. Consequently, daily consumption of the control group was the lowest with a mean value of 97.54 g DM/d compared to 99.37 g DM/d for the CFSGLSCS group. To begin with the dietary effect of the whole fenugreek seed, Mustafa [44] reported that the dietary addition of the fenugreek seed at a level of 0.05%, 0.1% and 0.15% didn’t affect feed consumption of Hy-Line White layers during a 40–59 week of age. A similar finding to the previous was reported by Abdouli et al. [9] who showed that the ground fenugreek at two to six levels didn’t affect feed intake of 69 wk-old Lohman White laying hens. A study conducted by Nasra et al. [20] reported that the supplementation of 0.5% ground fenugreek on local Mandarah strain hens diets during their 16–28 weeks of age decreased feed consumption after eight weeks. By contrast, feed consumption increased after 12 weeks of treatment. Our data are in accordance with those reported by Ademola et al. [45] who found that garlic oil and cholestyramine increased feed intake of Black Harco laying hens. By contrast, Olobatoke and Mulugeta [46] showed that garlic powder at 3% or 4% reduced feed consumption of 30 weeks old Dekalb White laying birds. Sibel et al. [47] showed that feed consumption was not affected by the dietary supplementation of garlic powder during a 12-week period. Similar results have been reported by Safaa [16] who showed that the dietary incorporation of 2% of garlic powder or fenugreek seed did not affect the daily feed intake. Chowdhury et al. [48] showed that the dietary addition of 0% to 10% of garlic paste during a six week-period did not affect feed consumption. Concerning the linseed effect, Ahmad et al. [49] reported that feed intake of White Leghorn laying hens decreased with the increase of linseed level from 0% to 15%. However, Criste-Rodica et al. [50] found that feed intake was not affected by the dietary addition of 5% linseed from the 35th to 42nd week-old of Lohman Brown layers. The dietary effect of copper has been investigated by several studies. Idowu et al. [51] demonstrated that feed intake of 30 week-aged Black Harco layer strain increased when inorganic Cu (CuSO4.5H2O) was added for 10 weeks compared with Cu Proteinate. By contrast, Kaya et al. [52] showed that the dietary supplementation of 200 ppm of copper didn’t affect the feed intake of 38 week-old Lohman White layers. 

Similar results have been reported by Rahimi et al. [53] who found that the dietary addition of 15 g/kg of sun dried garlic powder, 200 mg/kg of cupric sulfate pentahydrate alone or together for 40 week-aged Single Comb White Leghorn (SCWL) laying hens didn’t affect feed consumption. Pekel et al. [34] showed that the Lohman Brown laying hens receiving 250 ppm of Cu decreased their feed consumption. Hens’ body weight loss was not affected (*p* > 0.05) by the dietary restriction. Body weight losses varied from 71.41 g to 120.86 g during the 42nd day. Similar data were found by Abdouli et al. [9] who reported that the Lohman White laying hens received 100 g of basal diet/d without or with fenugreek seed addition during 49 days had a body loss with a mean value of −115.3 g. In the present study, egg production and egg mass decreased for the laying hens fed on the experimental diet when compared to the control group. However, these changes were not statistically significant (*p* > 0.05) for egg mass. Feed efficiency increased (*p* > 0.05) for CFSGLSCS with a mean value of 1.98 compared to 1.94 for control. Our data were partially in accord with these of Aderemi et al. [54] who reported that the laying production and egg weight increased when garlic powder was incorporated at 4% garlic powder. Sibel et al. [47] showed that an increase of egg production was found when 0.5% and 1% of garlic powder was supplemented compared to the 2% garlic powder supplemented group. Hens receiving 1% of garlic powder had the highest egg production and egg weight when compared to those fed on 0%, 0.5%, and 2% of garlic powder. The previous author had attributed the negative effect of the 2% of garlic powder on feed consumption to the strong odor of garlic acting as a deterrent. According to Safaa [16], the dietary incorporation of 2% garlic or fenugreek did not affect egg rate, egg weight, egg mass and feed conversion. In agreement with the previous study, Chowdhury et al. [48] showed that egg production and feed efficiency was not affected by the dietary addition of 0% to 10% of garlic paste. The effect of linseed on laying performance has been reported by Ahmad et al. [49], Al-Nasser et al. [55] and Criste-Rodica et al. [50] who showed that the dietary addition of flaxseed didn’t affect egg production rate and weight. By contrast Krawczyk et al. [56] reported that the dietary supplementation of 10% of linseed increased egg weight. Pekel et al. [34] showed that laying hens fed on 250 ppm of Cu from Cu sulfate had the highest egg production mean value with the lowest egg weight. However Attia et al. [57] reported that 60 ppm of inorganic Cu increased egg weight and egg mass of laying hens. Rahimi et al. [53] reported that the dietary incorporation of garlic powder, Cu sulfate together or alone did not affect laying production, egg weight, egg mass and feed conversion ratio. 

### 3.2. Egg Physical Characteristics

Egg weight and physical characteristics are shown in Table 3. It seems clear that the dietary addition of CFSGLSCS increased (*p* < 0.05) egg and shell weight. This combination didn’t affect (*p* > 0.05) albumen weight, yolk weight and shell thickness. Our data were in agreement with these reported by Abdouli et al. [9] who showed that egg and shell weights of hens fed on water and hexane insoluble fraction of whole fenugreek seed were the highest when compared to the group fed on whole fenugreek seed. The effect of feeding garlic on the egg physical characteristics has been treated by Safaa [16] who found an increase of egg yolk percentages and a decrease of albumen weight percentages. According to Ahmad et al. [49], the dietary incorporation of linseed at a level of 0% to 15% did not affect egg quality characteristics. However, Pekel et al. [34] reported a decrease of egg shell thickness of laying hens fed on 250 mg/kg of Cu lysine.

### 3.3. Lipid Profile

Egg yolk cholesterol concentration reduction may be achieved by feeding laying hens with a diet containing special mixtures of fenugreek seeds, garlic and CuSO4. In fact, many medicinal properties attributed to fenugreek seed (*Trigonella foenum graecum* L.) [58,59,60], but there is scanty documented literature on its use to lower egg yolk cholesterol. However, Abdouli et al. [9], showed that the positive effect of ground fenugreek seeds on serum cholesterol concentrations would be due the particular composition of the saponins in fenugreek seed, or to an unknown synergetic effect of saponins and other bioactive compounds in fenugreek seed. Concerning copper, it is an essential mineral with a regulating activity of cholesterol biosynthesis by reducing the glutathione concentration. This co-enzyme decreases the mevalonate activity so that cholesterol one [61]. Two components of garlic, S-allylcysteine sulfoxide (alliin) and diallyl disulfide-oxide (allicin) were shown to lower cholesterol levels [62,63]. The overall effects of these constituents on lipid metabolism in chickens have not been described. Data of egg yolk triglycerides, lipids, total cholesterol and serum cholesterol are summarized in Table 4. Dietary treatment reduced egg yolk cholesterol (*p* < 0.05). Laying hens fed on CAFSLSSC had the lowest egg yolk cholesterol concentration with a mean value of 16.09 mg/g compared to 17.01 mg/g for the control group. However, the egg yolk cholesterol concentration decreased (*p* < 0.05) by 5.4% and egg weight increased (*p* < 0.05) from 55.18 g to 61.09 g. Consequently, the total egg cholesterol content was 240.98 mg/egg for the control group compared to 236.25 mg/egg for the experimental. These differences were not statistically significant (*p* > 0.05). Our results were in accordance with those reported by Safaa [16] who showed that the dietary supplementation of 2% of garlic or fenugreek decreased egg yolk cholesterol concentration to 7%. Ademola et al. [45] reported that garlic oil supplementation at 100 mg/kg reduced egg yolk cholesterol concentration when compared with hens’ given 200 mg/kg of garlic powder. A statistically significant decrease of egg yolk cholesterol concentration was also reported by Sibel et al. [47] who reported that the dietary addition of 0.5%, 1%, and 2% of garlic powder reduced egg yolk cholesterol concentration from 20.27 mg/g (control) to 13.21 mg/g (0.5%), 12.89 mg/g (1%) and 13.20 mg/g (2%). Dietary supplementation of garlic powder, copper sulfate or both decreased the egg yolk cholesterol concentration [53]. The latter reported that the hypocholesterolomic effect of 200 mg/kg of copper sulfate was more effective than 15 g/kg of garlic powder. According to Attia et al. [57], the dietary addition of Cu at 16 ppm and 120 ppm decreased egg yolk cholesterol with 9% and 13%, respectively. As well as the yolk cholesterol serum cholesterol decreased from 158.42 (C) mg/dL to 122.88 mg/dL (CFSGLSCS). A similar finding has been reported by Abdouli et al. [9] who showed that ground fenugreek seed at levels ranging from zero to six g/hen/d decreased serum cholesterol concentration from 106.4 mg/dL (control) to 85.8 mg/dL, 92.7 mg/dL, and 86.2 mg/dL for respectively, 2 g, 4 g, and 6 g. This decrease in the serum cholesterol concentration would be attributable to phenols, saponins and other bioactive compounds of the fenugreek seed. Saponins content of fenugreek seeds was 2.4%. According to Sauvaire et al. [64] diosgenin, the principal furostanol of fenugreek seeds may have a hypocholesterolemic effect on plasma cholesterol concentration. The mechanism of these reductions had been demonstrated that diosgenin is responsible for cholesterol absorption inhibition and thus reduces liver cholesterol concentration and consequently increases the cholesterol secretion of bile and the fecal excretion of neutral sterols. Chowdhury et al. [48] showed that garlic paste at 2%, 4%, 6%, 8% or 10% in laying hens diet reduced egg yolk cholesterol by 5%, 9%, 14%, 20% and 24%, respectively and leaded to a reduction of blood cholesterol by 15%, 28%, 33% and 43% for 2%, 4%, 6% and 8% of garlic paste. This author proposed that egg yolk and blood cholesterol reduction when hens were given garlic paste might be attributed to the reduction of synthetic enzyme activity. However, Attia et al. [57] demonstrated that the organic source of Cu decreased plasma cholesterol by 12.8% compared to 9.2% inorganic source and confirmed the hypothesis of Kim et al. [61] who reported that higher levels of Cu depressed hepatic glutathione formation thus the cholesterol one.

## 4. Conclusions

The results of the present studies clearly showed that fenugreek seeds (3%), garlic (1%), linseeds (3%) and copper sulfate (0.078%) combination is a primary factor causing significant reduction of egg yolk cholesterol concentration (from 17.01 mg/g to 16.09 mg/g yolk) and blood cholesterol (from 158.42 g/egg to 122.88 g/egg) without imposing any adverse effect on performances parameters. However, response to the dietary incorporation of linseed in terms of the fatty acids profile needs to be evaluated. 

## Figures and Tables

**Table 1:** Ingredients and calculated offered components of experimental diets.

Diets		
Ingredients	Control (C)	Experimental (CGFSLSSC)
Yellow corn	61	59.92
Soybean meal	22	21
Calcium carbonate	8	8
Mineral and vitamin mixture ^§^	4	4
Basal Diet, kg	95	92.92
Whole fenugreek seeds(WFS), kg	0	2.9
Garlic paste, g	0	970
Whole Linseeds (WLS), kg	0	2.9
CuSO4, g	0	76
Vegetable Oil, kg	5	0
Total	100	100
Chemical Composition, % Dry Matter		
Dry Matter	91.56	90.35
Ash	21.76	23.62
Crude Protein	16.54	17.27
Ether Extract	3.35	7.91
NDF	10.15	13.00
Hemi-cellulose	6.98	8.92
^¥^ Metabolizable Energy, kcal/kg DM	2741.1	2962.7

Note: ^§^ Control provided following nutrients per 100 g: Ca, 4.3 g; P, 0.6 g; Na, 0.14 g; Cl, 0.23 g; Fe, 4 mg; Zn, 40 mg; Mn, 7 mg; Cu, 0.3 mg; I, 0.08 mg; Se, 0.01 mg; Co, 0.02; methionine,0.39 g; methionine + cysteine, 0.69 g; lysine, 0.89 g; Retinol, 800 IU; Cholecalciferol, 220 IU; α-tocopherol, 1.1 IU; Thiamin, 0.33 IU; Nicotinic acid, 909 IU. NDF: Neutral detergent fiber. ^¥^ Metabolizable Energy = 2707.71 + 58.63*EE 16.06*NDF [39].

**Table 2:** Effects of various diets on the laying hen performances.

Parameters	Diets		Statistics	
	Control (C)	CFSGLSCS	SEM	*p*-Value
Daily refusal (g DM/d)	2.46 ^a^ ± 3.97	0.62 ^b^ ± 1.33	0.26	<0.0001
Feed consumption (g DM/d)	97.54 ^b^ ± 3.97	99.37 ^a^ ± 1.34	0.25	<0.0001
Hen-day laying rate (%)	90.84 ^a^ ± 11.89	87.89 ^b^ ± 10.95	0.99	<0.036
Body weight change (g)	−71.41 ± 109.23	−120.86 ± 112.23	23.61	0.146
Egg mass (g/hen/d)	50.95 ± 3.61	50.87 ± 6.19	0.53	0.92
Feed efficiency	1.94 ± 0.27	1.98 ± 0.3	0.02	0.19

Note: C = control; CFSGLSCS = diet contained: 2.9 kg of fenugreek seed, 2.9 kg of linseed, 76 g of CuSO_4_ and 970 g of garlic mixed with 92 kg of basal diet; SEM = Standard Error of the mean; ^a,b^: Mean in the same row having different superscripts are significantly different (*p* < 0.05).

**Table 3:** Effects of various diets on egg characteristics.

	Diets		Statistics	
	Control (C)	CFSGLSCS	SEM	*p*-Value
Egg weight (g)	55.18 ^b^ ± 3.99	61.09 ^a^ ± 3.36	0.62	0.0058
Shell weight (g)	6.12 ^b^ ± 0.56	6.58 ^a^ ± 0.44	0.08	0.0001
Shell thickness (mm)	0.39 ± 0.035	0.4 ± 0.04	0.0058	0.32
Albumen weight (g)	32.83 ^b^ ± 3.11	34.12 ^a^ ± 2.9	0.48	0.058
Yolk weight (g)	14.74 ^b^ ± 0.93	15.24 ^a^ ± 1.32	0.18	0.056

Note: C = control; CFSGLSCS = diet contained: 2.9 kg of fenugreek seed, 2.9 kg of linseed, 76 g of CuSO_4_ and 970 g of garlic mixed with 92 kg of basal diet; SEM = Standard Error of the mean; ^a,b^: Mean in the same row having different superscripts are significantly different (*p* < 0.05).

**Table 4:** Effects of various diets on lipids profile.

	Diets		Statistics	
	Control (C)	CFSGLSCS	SEM ^δ^	*p*-Value
Triglyceride, mg/g yolk	202.2 ± 14.94	213.74 ± 19.4	5.22	0.13
Triglyceride, mg/egg	2859.14 ± 234.9	3143.43 ± 234.9	100.38	0.59
Total cholesterol, mg/g yolk	17.01 ^a^ ± 1.1	16.09 ^b^ ± 0.73	0.28	0.0317
Total cholesterol, mg/egg	240.98 ± 22.4	236.25 ± 19.00	6.33	0.60
Total lipids (g/egg)	4.59 ^b^ ± 0.45	5.23 ^a^ ± 0.46	0.138	0.0038
Serum cholesterol (mg/dL)	158.42 ^a^ ± 32.1	122.88 ^b^ ± 24.2	10.55	0.0242

Note: C = control; CFSGLSCS = diet contained: 2.9 kg of fenugreek seed, 2.9 kg of linseed, 76 g of CuSO_4_ and 970 g of garlic mixed with 92 kg of basal diet; ^δ^ SEM = Standard Error of the mean; ^a,b^: Mean in the same row having different superscripts are significantly different (*p* < 0.05).

## References

[1] Santini A., Tenore G.C., Novellino E. (2017). Nutraceuticals: A paradigm of proactive medicine. Eur. J. Pharm. Sci..

[2] Daliu P., Santini A., Novellino E. (2018). A decade of nutraceutical patents: Where are we now in 2018?. Expert Opin. Ther. Pat..

[3] Durazzo A., D’Addezio L., Camilli E., Piccinelli R., Turrini A., Marletta L., Marconi S., Lucarini M., Lisciani S., Gabrielli P. (2018). From plant compounds to botanicals and back: A current snapshot. Molecules.

[4] Durazzo A., Lucarini M. (2019). Extractable and non-extractable antioxidants. Molecules.

[5] Durazzo A., Lucarini M., Souto E.B., Cicala C., Caiazzo E., Izzo A.A., Novellino E., Santini A. (2019). Polyphenols: A concise overview on the chemistry, occurrence and human health. Phyt. Res..

[6] Meluzzi A., Sirri F., Manfreda G., Tallarico N., Franchini A. (2000). Effects of dietary vitamin E on the quality of table eggs enriched with n-3 long-chain fatty acids. Poult. Sci..

[7] Anton M., Nau F., Nys Y. (2006). Bioactive egg components and their potential uses. Wourld’s Poult. Sci. J..

[8] Naviglio D., Gallo M., Le Grottaglie L., Scala C., Ferrara L., Santini A. (2012). Determination of cholesterol in Italian chicken eggs. Food Chem..

[9] Abdouli H., Belhouane S., Hcini E. (2014). Effect of fenugreek seeds on hens’ egg yolk color and sensory quality. J. New Sci..

[10] Abdouli H., Omri B., Tayachi L. (2014). Effect of whole fenugreek seed before and after its maceration in water on hens’ laying performance and egg cholesterol profile. J. New Sci..

[11] Weggemans R.M., Zock P.L., Katan M.B. (2001). Dietary cholesterol from eggs increases the ratio of total cholesterol to high-density lipoprotein cholesterol in humans: A meta-analysis. Am. J. Clin. Nutr..

[12] Omri B., Mourou I., Abdouli H., Tayachi L. (2015). Effect of fenugreek seed supplementation on hens’ egg fatty acids profile and atherogenic and thrombogenic health lipid indices. J. New Sci..

[13] Lamas A., Anton X., Miranda J.M., Roca-Saavedra P., Cardelle-Cobas A., Rodriguez J.A., Franco C.M., Cepeda A. (2016). Technological development of functional egg products by an addition of n-3 polyunsaturated-fatty-acid-enriched oil. CyTA-J. Food.

[14] Aida H., Hamandiz M., Gagic A., Mihaljevic M., Krinic J. (2005). Egg yolk lipid modifications by fat supplemented diets of laying hens. Acta Vet..

[15] Rahardja D.P., Hakim M.R., Pakiding W., Lestari V.S. (2010). Hypocholesterolemic effect of garlic powder in laying hen: Low cholesterol egg. J. Ind. Trop. Anim. Agric..

[16] Safaa H.M. (2007). Effect of dietary garlic or fenugreek on cholesterol metabolism in laying hens. Egypt Poult. Sci..

[17] Bakalli R.I., Pesti G.M., Ragland W.L., Konjofca V. (1995). Dietary copper in excess of nutritional requirement reduces plasma and breast muscle cholesterol of chickens. Poult. Sci..

[18] Pesti G.M., Bakalli R.I. (1998). Studies on the effect of feeding cupric sulfate pentahydrate to laying hens on egg cholesterol content. Poult. Sci..

[19] Caston L.J., Squires E.J., Lesson S. (1994). Hen performance, egg quality and the sensory evaluation of eggs from SCWL hens fed dietary flax. Can. J. Anim. Sci..

[20] Nasra B.A., Yahya Z.E., Abd El-Ghany F.A. (2010). Effect of dietary supplementation with phytoestrogens sources before sexual maturity on productive performance of Mandarah hens. Egypt Poult. Sci..

[21] Bean L.D., Leeson S. (2003). Long-term effects of feeding flaxseed on performance and egg fatty acid composition of brown and white hens. Poult. Sci..

[22] Quershi A.A., Din Z.Z., Buirmeileh N., Burger W.C., Ahmad Y., Elson C. (1983). Suppression of avian hepatic lipid metabolism by solvent extracts of garlic: Impact on serum lipids. J. Nutr..

[23] Sharma R.K., Singh R.A., Pal R.N., Aggarwal C.K. (1979). Cholesterol content of chicken eggs as affected by feeding garlic, sarpagandha, and nicotinic acid. Haryana Agriculture University. J. Res..

[24] Botsoglou N.A., Yannakopoulos A.L., Fletouris D.J., Tserveni-Goussi A.C., Psomas I. (1998). Yolk fatty acid composition and cholesterol content in response to level and form of dietary flaxseed. J. Agric. Food Chem..

[25] Caston L., Leeson S. (1990). Dietary flaxseed and egg composition. Poult. Sci..

[26] Sim J.S., Carter J.R. (1990). Flaxseed as a high energy/protein/omega-3 fatty acid ingredient for poultry. Proceedings of the 53rd Flax Institute of the United States.

[27] Jiang Z., Ahn D.U., Ladner L., Sim J.S. (1992). Influence of feeding full fat flax and sunflower seeds on internal and sensory qualities of eggs. Poult. Sci..

[28] Sari M., Aksit M., Ӧzdogan M., Basmacıoglu H. (2002). Effects of addition of flaxseed to diets of laying hens on some production characteristics, levels of yolk and serum cholesterol, and fatty acid composition of yolk. Arch. Für Geflügelkunde.

[29] Mabrouki S., Omri B., Abdouli H. (2015). Chemical, functional and nutritional characteristics of raw, autoclaved and germinated fenugreek seeds. J. New Sci..

[30] Omri B., Chalghoumi R., Abdouli H. (2017). Effect of unprocessed, autoclaved and pre-germinated fenugreek seeds on hens ‘laying performance, egg quality characteristics and chemical composition’. Rev. Colomb. Cienc. Pecu..

[31] Yeh Y.Y., Liu L. (2001). Cholesterol-lowering effect of garlic extracts and organosulfur compounds: Human and animal studies. J. Nutr..

[32] Motamedi S.M., Taklimi S.M.M. (2014). Investigating the effect of fenugreek seed powder and garlic powder in the diet on immune response of commercial laying hens’ egg. Indian J. Sci. Res..

[33] Adebiyi F.G., Ologhobo A.D., Adejumo I.O. (2018). Raw *Allium sativum* as performance enhancer and hypocholesterolemic agent in laying hens. Asian J. Anim. Vet. Adv..

[34] Pekel A.Y., Alp M. (2011). Effects of different dietary copper sources on laying hen performance and egg yolk cholesterol. J. Appl. Poult. Res..

[35] Jegede A.V., Oso A.O., Fafiolu A.O., Sobayo R.A., Idowu O.M.O., Oduguwa O.O. (2015). Effect of dietary copper on performance, serum and egg yolk cholesterol and copper residues in yolk of laying chickens. Slovak J. Anim. Sci..

[36] Omri B., Chalghoumi R., Abdouli H. (2017). Study of the Effects of Dietary supplementation of linseeds, fenugreek seeds and tomato-pepper mix on laying hen’s performances, egg yolk lipids and antioxidants profiles and lipid oxidation status. J. Anim. Sci. Livest. Prod..

[37] Omri B., Chalghoumi R., Izzo L., Ritieni A., Lucarini M., Durazzo A., Abdouli A., Santini A. (2019). Effect of dietary incorporation of linseed alone or together with tomato-red pepper mix on laying hens’ egg yolk fatty acids profile and health lipid indexes. Nutrients.

[38] Yassein S.A., El-Mallah G.M., Sawsan M.A., El-Ghamry A.A., Abdel-Fattah M.M., El-Harriry D.M. (2015). Response of laying hens to dietary flaxseed levels on performance, egg quality criteria, fatty acid composition of egg and some blood parameters. Int. J. Res. Stud. Biosci..

[39] Nascimento G.A.J. (2007). Prediction Equations of the Energetic Values of Poultry Feedstuffs for Utilizing the Meta-Analysis Principle. Ph.D. Thesis.

[40] Van Soest P.J., Robertson J.B., Lewis B.A. (1991). Methods of dietary fiber, neutral detergent fiber and non-starch carbohydrates in relation to animal nutrition. J. Dairy Sci..

[41] AOAC (1984). Association of Official Analytical Chemists. Official Methods of Analysis.

[42] Pasin G., Smith G.M., O’Mahony M. (1998). Rapid determination of total cholesterol in egg yolk using commercial diagnostic cholesterol reagent. Food. Chem..

[43] SAS (1989). Statistical Analysis System.

[44] Moustafa K.K. (2006). Effect of using commercial and natural growth promoters on the performance of commercial laying hens. Egypt. J. Poult. Sci..

[45] Ademeola S.G., Sikiru A.B., Akinwumi O., Olamiyi O.F., Egbewande O.O. (2011). Performance, yolk lipid, egg organoleptic properties and haematological parameters of laying hens fed cholestryamine and garlic oil. Glob. Vet..

[46] Olobatoke R.Y., Mulugeta S.D. (2011). Effect of dietary garlic powder on layer performance, fecal bacterial load, and egg quality. Poult. Sci..

[47] Sibel C., Mesut K., Zeynep E., Mikail B., Altug K., Vesile D., Ali K.O. (2009). Effect of galiric powder on egg yolk and serum cholesterol and performances of laying hens. Bull. Vet. Inst. Pulawy.

[48] Chowdhury S.R., Chowdhury S.D., Smith T.K. (2002). Effects of dietary garlic on cholesterol metabolism in laying hens. Poult Sci..

[49] Ahmad S., Ahsan H., Yousaf M., Kamran Z., Ata R., Sohail M.U., Shahid R. (2013). Effect of feeding whole linseed as a source of polyunsaturated fatty acids on performance and egg characteristics of laying hens kept at high ambient temperature. Braz. J. Poult. Sci..

[50] Criste-Rodica D., Tudora-Dumitra P., Ciurescu C., Ropota M., Rachieru D. (2009). Effects of moderate (5%) levels of linseed in layer diets. Arch. Zootech..

[51] Idowu O.M.O., Laniyan T.F., Kuye O.A., Oladele-Ojo V.O., Eruvbetine D. (2006). Effect of Copper Salts on performance, cholesterol, residues in liver, eggs and excreta of laying hens. Arch. Zootech..

[52] Kaya A., Kaya H., Macit M., Çelbi Ş., Esenbuğa N., YőrűK M.A., Karaoğlu M. (2013). Effects of Dietary Inclusion of Plant Extract Mixture and Copper into Layer Diets on Egg Yield and Quality, Yolk Cholesterol and Fatty Acid Composition. Kafkas Univ. Vet. Fak. Derg..

[53] Rahimi S.h., Rafiei A., Lotfollahian H., Afsharnaderi A. (2008). Influence of combined usage of garlic powder and copper on egg yolk cholesterol concentration in laying hens. J. Vet. Res..

[54] Aderemi F., Olufemi A., Oluseyi A. (2013). Evaluating pepper (Capsicum annuum) and garlic (*Allium sativum*) on performance egg trait and serum parameters of old layers. J. Biol. Agric. Healthc..

[55] Al-Nasser A.Y., Al-Saffar A.E., Abdullah F.K., Al-Bahouh M.E., Gehan R., Magdy M.M. (2011). Effect of adding flaxseed in the diet of laying hens on both production of omega-3 enriched eggs and on production performance. Int. J. Poult. Sci..

[56] Krawczyk J., Bzducha E.S., Chomentowska Z.K., Semik E. (2011). Efficiency of feeding linseed to heritage breeds hens. Ann. Anim. Sci..

[57] Attia Y.A., Abdalah A.A., Zeweil H.S., Bovera F., Tag El-Din A.A., Araft M.A. (2011). Effect of inorganic or organic copper additions on reproductive performance, lipid metabolism and morphology of organs of dual-purpose breeding hens. Arch. Geflugelk..

[58] Basu S.K. (2006). Seed Production Technology for Fenugreek (*Trigonella foenumgraecum* L.) in the Canadian Prairies. Master’s Thesis.

[59] Acharya S.N., Srichamroen A., Basu S.K., Ooraikul B., Basu T. (2006). Improvement in the nutraceutical properties of fenugreek (*Trigonella foenum-graecum* L.). Songklanakarin J. Sci. Technol..

[60] Acharya S.N., Thomas J.E., Basu S.K. (2008). Fenugreek (*Trigonella foenum-graecum* L.) an alternative crop for semiarid regions of North America. Crop. Sci..

[61] Kim J.W., Chao P.Y., Allen A. (1992). Inhibition of elevated hepatic glutathione abolishes copper deficiency cholesterolemia. FASEB J..

[62] Itokawa Y., Inoue K., Sasagawa S., Fujiwara M. (1973). Effect of S-methylcysteine sulfoxide, S-allylcysteine sulfoxide and related sulfur-containing amino acids on lipid metabolism of experimental hypercholesterolemic rats. J. Nutr..

[63] Ali M., Al-Qattan K.K., Al-Enezi F., Khanafer R.M., Mustafa T. (2000). Effect of allicin from garlic powder on serum lipids and blood pressure in rats fed with a high cholesterol diet. Prostaglandins Leukot. Essent. Fatty Acids.

[64] Sauvaire Y., Ribes G., Baccou J.C., Loubatieères-Mariani M.M. (1991). Implication of steroid saponins and sapogenins in the hypocholesterolemic effect of fenugreek. Lipids.

